# East Africa International Center of Excellence for Malaria Research: Impact on Malaria Policy in Uganda

**DOI:** 10.4269/ajtmh.21-1305

**Published:** 2022-10-13

**Authors:** Jane F. Namuganga, Joaniter I. Nankabirwa, Catherine Maiteki-Ssebuguzi, Samuel Gonahasa, Jimmy Opigo, Sarah G. Staedke, Damian Rutazaana, Chris Ebong, Grant Dorsey, Sheena S. Tomko, Timothy Kizza, Henry D. Mawejje, Emmanuel Arinaitwe, Philip J. Rosenthal, Moses R. Kamya

**Affiliations:** ^1^ Infectious Diseases Research Collaboration, Kampala, Uganda;; ^2^ Makerere University College of Health Sciences, Kampala, Uganda;; ^3^ National Malaria Control Division, Ministry of Health, Kampala, Uganda;; ^4^ London School of Hygiene and Tropical Medicine, London, United Kingdom;; ^5^ Department of Medicine, University of California San Francisco, San Francisco, California;; ^6^ Department of Biology, University of Pennsylvania, Philadelphia, Pennsylvania

## Abstract

Malaria is the leading cause of disease burden in sub-Saharan Africa. In 2010, the East Africa International Center of Excellence for Malaria Research, also known as the Program for Resistance, Immunology, Surveillance, and Modeling of Malaria (PRISM), was established to provide a comprehensive approach to malaria surveillance in Uganda. We instituted cohort studies and a robust malaria and entomological surveillance network at selected public health facilities that have provided a platform for monitoring trends in malaria morbidity and mortality, tracking the impact of malaria control interventions (indoor residual spraying of insecticide [IRS], use of long-lasting insecticidal nets [LLINs], and case management with artemisinin-based combination therapies [ACTs]), as well as monitoring of antimalarial drug and insecticide resistance. PRISM studies have informed Uganda’s malaria treatment policies, guided selection of LLINs for national distribution campaigns, and revealed widespread pyrethroid resistance, which led to changes in insecticides delivered through IRS. Our continuous engagement and interaction with policy makers at the Ugandan Ministry of Health have enabled PRISM to share evidence, best practices, and lessons learned with key malaria stakeholders, participate in malaria control program reviews, and contribute to malaria policy and national guidelines. Here, we present an overview of interactions between PRISM team members and Ugandan policy makers to demonstrate how PRISM’s research has influenced malaria policy and control in Uganda.

## INTRODUCTION

Malaria remains one of the most important global health challenges, with an estimated 200 million clinical cases and more than 600,000 deaths in 2020, with most of the disease burden concentrated in sub-Saharan Africa.^1^ In Uganda, malaria is endemic in 95% of the country, with unstable malaria transmission in the remaining 5%. Malaria is the leading cause of morbidity and mortality in the country, accounting for 40% of outpatient visits and 11% of hospital deaths.^2^

Like many other countries in sub-Saharan Africa, Uganda has scaled up malaria control interventions, including the adoption of artemether-lumefantrine (AL) as first-line treatment of malaria in 2004 and repeated distribution of long-lasting insecticidal nets (LLINs) through both routine distribution channels and universal coverage campaigns in 2013–2014, 2017–2018, and 2020–2021. In addition, indoor residual spraying of insecticide (IRS) was reinitiated in Uganda in 2006 after a gap of 40 years and is currently being implemented in 14 of 135 districts.^3^ Monitoring the impact of these interventions on the malaria disease burden is routinely conducted through Demographic Health Surveys, and Malaria Indicator Surveys, and the country’s routine Health Management Information System (HMIS). Despite their benefits, national surveys are costly and infrequent, making it difficult to use their results for day-to-day decision-making.

The Program for Resistance, Immunology, Surveillance, and Modeling of Malaria (PRISM) was established in 2010, with funding from the U.S. National Institutes of Health, to provide comprehensive malaria surveillance and measure the impact of population-level malaria control interventions.^4^ We present here how PRISM data collected over the past 11 years has been used to improve the evidence base for malaria, estimate the impact of population-level control interventions, and guide responses to challenges to malaria control in Uganda.

## USING THE PRISM PLATFORM TO INTERACT WITH POLICY MAKERS AND INFORM IMPLEMENTATION OF MALARIA INTERVENTIONS

### Translation of research to policy.

Meaningful translation of research into policy requires engagement with stakeholders throughout the policy development cycle (formulation, implementation, and evaluation). The PRISM team has continuously engaged and interacted with the Ministry of Health (MoH) National Malaria Control Division (NMCD) and other stakeholders through several avenues, including participation in technical working group meetings, midterm and end-term review workshops, and weekly partner coordination meetings. The team has also led four malaria research-to-policy dissemination meetings in 2016, 2017, 2018, and 2019 at which PRISM research findings were disseminated to the malaria research and policy community in Uganda. Key meeting participants have included NMCD officials; district health teams; development and technical partners from the World Health Organization and U.S. Agency for International Development–President’s Malaria Initiative; malaria implementing partners including Malaria Consortium, Pilgrim Africa, Clinton Health Access Initiative, UNICEF, and the Program for Accessible Health and Communication; as well as interested Ugandan researchers and students. In addition, quarterly PRISM malaria surveillance reports are shared with malaria stakeholders. Engagement in these different forums have allowed sharing of data from PRISM and collaborating groups, including outcomes of the evaluation of malaria epidemiology and control interventions such as impacts of the universal LLIN distribution campaigns,^5^^,^^6^ impacts of starting or stopping IRS in different districts,^7^ treatment efficacy of antimalarial drugs,^8^ and insecticide resistance patterns, which have guided national malaria control efforts.

### LLINs.

PRISM cohort studies demonstrated limited impact of the 2013–2014 LLIN distribution campaign on malaria disease burden at three sites with varying transmission intensities—an outcome attributed to the emergence of pyrethroid resistance in anopheline mosquitoes.^9^ These findings informed the use of LLINs containing the synergist piperonyl butoxide (PBO) in selected districts during the 2017–2018 campaign. PRISM also provided the infrastructure and expertise required for a cluster-randomized trial (LLINEUP) to evaluate the effects of pyrethroid LLINs with and without PBO on malaria indicators in 48 districts in Uganda embedded in the 2017–2018 national LLIN distribution campaign.^5^^,^^6^ This trial showed that PBO LLINs reduced parasite prevalence more effectively than conventional LLINs for up to 25 months post-distribution.^5^ These findings influenced the Ugandan NMCD decision to deliver PBO LLINs in the 2020–2021 national LLIN distribution campaign and generated data for the WHO Vector Control Advisory group on the public health impact of PBO LLINs, supporting WHO’s recommendation for use of PBO LLINs in areas where pyrethroid resistance is high. PRISM is now conducting a second cluster-randomized trial (LLINEUP2) to compare the impacts of a new dual active-ingredient LLIN (Disease Control Technologies [DCT], USA, Royal Guard^®^, treated with alpha-cypermethrin and pyriproxyfen) with a PBO LLIN, embedded in Uganda’s 2020–2021 LLIN distribution campaign. In addition, PRISM studies have demonstrated high LLIN attrition rates, an important issue to address in future campaigns,^10^ as LLINs will continue to be a cornerstone of malaria control in Uganda.^11^ More research is needed to gain a better understanding of the factors that influence LLIN use, adherence, and effectiveness in high malaria transmission settings and in areas with high levels of insecticide resistance.

### Malaria Reference Centers.

Malaria Reference Centers (MRCs) are established at high-volume, level III/IV public health facilities located throughout Uganda in areas with varying malaria transmission intensities. MRCs were first established in 2006 by the Uganda Malaria Surveillance Project (UMSP), a project led by Makerere University; the University of California, San Francisco; and the Infectious Diseases Research Collaboration, in collaboration with the Uganda MoH.^12^ There are currently 70 MRCs collecting high-quality, individual-level data from all patients presenting to outpatient clinics of these facilities. MRCs and cohort studies have provided opportunities to analyze large datasets collected over 15 years. MRC data were used to assess the impact of starting and subsequently stopping IRS in various districts on malaria burden in northern and eastern Uganda. IRS greatly reduced the malaria burden when rolled out in high-transmission settings.^7^^,^^9^^,^^13^ However, stopping IRS resulted in increases in the malaria burden to almost pre-IRS levels within a few months.^7^ Our cohorts provided more detailed data on the impacts of IRS on malaria transmission, infection, and disease in the adjacent Tororo (with IRS) and Busia (without IRS) districts in eastern Uganda.^13^^,^^14^ These data informed the response to malaria resurgences after IRS withdrawal and provided information on appropriate IRS coverage, intervals, insecticides, and discontinuation strategies.^15^ Most recently, we used MRC data to evaluate the impact of the COVID-19 epidemic on routine malaria indicators in Uganda from March 2020 to June 2021. We identified a reduction in the proportion of suspected malaria cases tested by rapid diagnostic tests, but COVID-19 did not otherwise appear to significantly impact routine malaria indicators in rural Uganda.^16^

### Drug efficacy and resistance.

Our group’s antimalarial drug efficacy and resistance studies have informed malaria prevention and treatment policies in Uganda.^8^^,^^17^^–^^22^ Our earlier studies reported high rates of treatment failure with chloroquine, sulfadoxine-pyrimethamine, and the combination of chloroquine plus sulfadoxine-pyrimethamine, which was the national treatment regimen from 2000 to 2004.^21^^–^^25^ These findings led to a change in the national recommendation for the treatment of uncomplicated malaria from chloroquine plus sulfadoxine-pyrimethamine to the ACT artemether-lumefantrine.^26^ After introduction of ACTs, we have conducted several studies to assess the efficacy and safety of first and second-line antimalarials. Our results have shown genotype-corrected efficacies for the ACTs artemether-lumefantrine, artesunate-amodiaquine, and dihydroartemisinin-piperaquine above 90%, the WHO threshold for consideration of a change in treatment guidelines.^17^^–^^20^^,^^27^ PRISM studies have shown no clear evidence of resistance to piperaquine or lumefantrine—findings that have supported the continued use of AL as first line and the establishment of dihydroartemisinin-piperaquine as second line treatment of uncomplicated malaria.^28^^–^^31^ Recently, there is growing concern about the emergence of artemisinin resistance in Uganda. We have seen increasing prevalence of two K13 propeller domain mutations (469Y and 675V), which were associated with artemisinin resistance in southeast Asia, in parasites collected at a number of sites in northern Uganda.^27^^,^^28^ Another group working in northern Uganda also identified these mutations and saw association between the mutations and artemisinin resistance (delayed clearance in vitro or after therapy).^32^^,^^33^ These results highlight the need for regular surveillance for markers of drug resistance across Uganda, a core component of the PRISM program. In addition, we continue to participate in therapeutic efficacy studies and studies evaluating new regimens for malaria chemoprevention in children and pregnant women.^34^^–^^36^ Continued efficacy studies will be essential to measure the impacts of artemisinin resistance on the treatment efficacies of ACTs.

### Insecticide resistance.

Coverage of vector control interventions in Africa has increased markedly, mainly through mass distribution of LLINs and targeted IRS.^37^ This scale-up of interventions has increased insecticide pressure on vector populations, selecting for resistant phenotypes.^38^ Insecticide resistance, particularly to pyrethroids, has spread across Africa, leading to fixation of resistant genes in some areas.^39^ Although the association between insecticide resistance and malaria disease burden largely remains unclear,^40^ high levels of pyrethroid resistance lead to a decline in LLIN effectiveness^38^ and an increase in *Plasmodium falciparum* sporozoite infection.^41^ Through the PRISM project, we have monitored insecticide resistance, demonstrating ubiquitous pyrethroid resistance and partial restoration of susceptibility to pyrethroids by the synergist PBO.^6^^,^^9^ In addition, reports of carbamate resistance contributed to the decision to change the IRS insecticide formulation from bendiocarb (2014–2015) to the organophosphate pirimiphos-methyl (2016–2019).^42^ More broadly, PRISM has contributed to mapping of insecticide resistance in Africa.^43^

## SUPPORT TO MALARIA CONTROL EFFORTS THROUGH ENHANCED HEALTH FACILITY–BASED MALARIA SURVEILLANCE IN UGANDA

Pillar three of the WHO Global Technical Strategy for malaria 2016–2030 proposed the transformation of surveillance into a core intervention for malaria control in areas of high endemicity.^44^ To adopt this strategy, Uganda included use of comprehensive surveillance as one of the pillars in the Uganda Malaria Reduction and Elimination Strategic Plan.^45^ HMIS is the primary source of malaria surveillance data in Uganda. In this system, aggregate data from all government-run and some private health facilities are assembled and reported at regular intervals using standardized registers and reporting forms. The introduction of the District Health Information System 2 (DHIS2), an electronic form of HMIS, in 2012 improved the collation of data at both district and national levels. However, despite these improvements, the quality of HMIS data remains limited by incomplete reporting, data entry errors, and a reliance on aggregate data.

**Figure 1.**
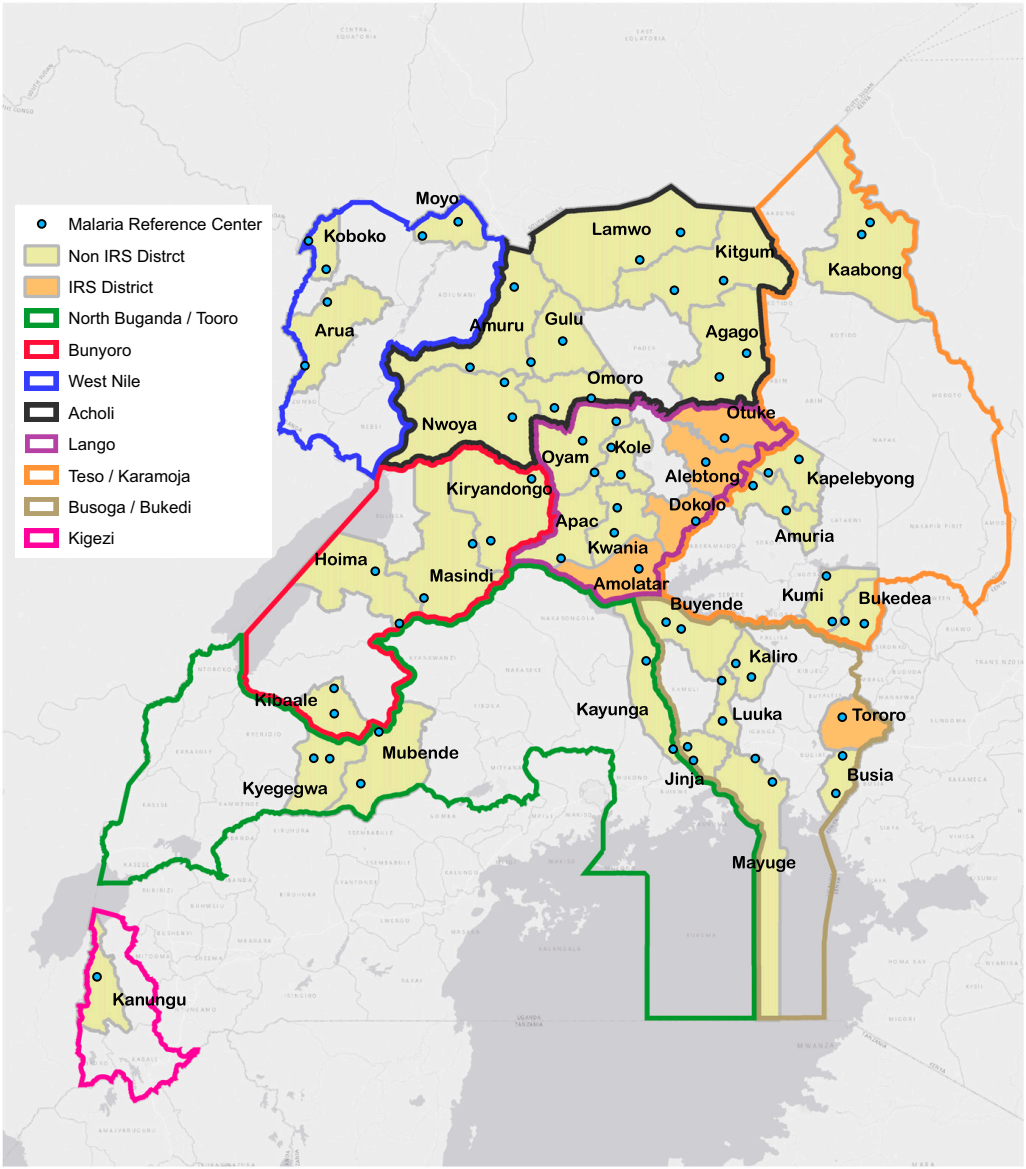
Map of Uganda with locations of sites where health facility–based malaria surveillance is being conducted.

PRISM’s UMSP project, which initially included six health facilities (MRCs) in 2006, has expanded to 70 facilities located in areas of varying malaria transmission intensity (Figure 1). MRC surveillance work has supported the transition from paper-based to electronic HMIS data management at health facilities. We have demonstrated that despite the challenges surrounding digitization of routine HMIS data in low-resource settings, including low levels of computer literacy, poor Internet connectivity, and unstable power supply at rural facilities, electronic data collection from primary health service delivery points is feasible. UMSP successfully collects routine data transcribed from paper-based HMIS forms into a user-friendly Access database. Health information assistants (HIAs), previously computer illiterate, have been trained to become model data managers, generating high-quality data, mentoring other HIAs within districts, and cascading best practices to other facilities. Furthermore, NMCD uses UMSP data to benchmark and validate malaria upsurges within MRC districts. The UMSP Access database allows for auto-generation of weekly and monthly HMIS reports for numerous variables in addition to malaria metrics. As such, reporting is easier and data accuracy significantly improved compared with nonelectronic systems. Training in malaria surveillance is linked to training in malaria case management and overall HMIS to improve outcomes. Data analysis at the facility level by HIAs has also been instituted at all MRCs by incorporating malaria dashboards into the UMSP electronic databases. External quality assurance for malaria microscopy is routinely done and has resulted in consistent adherence to the WHO malaria “test and treat” policy, with nearly 100% testing of patients suspected to have malaria at MRCs. The proportion of patients with a negative malaria test result prescribed an antimalarial is consistently less than 5%.^7^^,^^16^^,^^46^^–^^48^

In 2019, UMSP supplied reusable malaria normal channel and ACT stock monitoring charts to 70 MRCs. HIAs and facility in-charges were trained to plot and update these charts weekly, use them to monitor trends in malaria cases, identify any upsurges in malaria cases, monitor ACT stock status, and regularly provide updates to the district leadership and NMCD. ACT stock monitoring charts have facilitated identification of overstocked or understocked ACTs, guiding redistribution of excess drugs to other facilities and appropriate ordering of new stocks. Although the bulk of medicines and commodities are supplied by the MoH, our programs provide buffer stocks of malaria slides, reagents, and testing kits, and microscope repair and replacement, bridging supply chain gaps where feasible. Furthermore, analysis results from MRC data are triangulated with aggregate data from DHIS2 to confirm trends in malaria cases, report malaria upsurges, and impact control interventions. Data dissemination is undertaken by e-mail and through monthly data dissemination meetings held at each MRC.

The MRCs have provided a rich platform for monitoring trends in malaria burden and quantifying the impacts of malaria control interventions. The LLIN mass campaign strategy, as proposed by Yeka et al. in 2012,^4^ has materialized into three serial universal LLIN coverage campaigns since 2013, and NMCD has provided platforms and collaborated with PRISM to ensure evaluation of these campaigns.^6^^,^^49^ The LLINEUP and LLINEUP2 cluster-randomized trials provide substantive evidence that NMCD values and supports use of locally generated research findings to inform planning and design of each universal LLIN campaign.^6^^,^^49^ Preliminary work done in preparation for the LLINEUP2 study entailed demonstration of the feasibility of using routine health facility–based surveillance data to generate malaria incidence estimates.^48^ UMSP data collected at the MRC level can be modeled to evaluate and inform deployment of other malaria control interventions, including vaccine rollout and chemoprevention, and has provided a platform to compare data from research trials, with longitudinal routine health facility–based surveillance data providing a wide evidence base for malaria policy and program planning in Uganda.

## USING PRISM FOR CAPACITY DEVELOPMENT

In addition to informing policy and practice, PRISM provides a rich environment for research training in Uganda. PRISM has supported several local and international students engaged in degree and nondegree conferring training programs, allowing them to carry out independent research. Trainees have been supported primarily by the Fogarty International Center, leveraging the cohort and health facility–based PRISM infrastructure and the mentorship of senior PRISM scientists. Participation in key malaria program activities including midterm and end-term malaria program reviews, development of the malaria strategic plan, setting of the country’s malaria research agenda, and writing of Global Fund grant applications has provided a unique opportunity for PRISM team members to share data and expertise for policy decisions. At MRCs, the PRISM project facilitates skills transfer from project personnel to health workers at public health facilities to foster capacity building and sustainability, including on-site mentorship and training in malaria case management.

## SHARING PRISM DATA TO INCREASE IMPACT

Data sharing increases the reach and impact of PRISM studies by allowing others to reuse the data to achieve their own objectives. Data from the PRISM studies have been shared on https://clinepidb.org, https://plasmodb.org, and https://vectorbase.org (Table 1). Making data publicly accessible on these platforms has decreased the burden of providing data for the data management team. For example, collaborators were able to identify blood samples of interest based on associated clinical data available via ClinEpiDB without assistance from PRISM staff. In addition, external research groups have been able to further their own research using publicly accessible data. For example, PRISM data were used to inform a model to distinguish between the probabilities of malaria-attributable fever and nonmalarial febrile illness in children in sub-Saharan Africa.^50^ Finally, platforms like ClinEpiDB and VectorBase make it easier for people without a strong background in data analytics to visualize and interpret data. This can be particularly important in helping policy makers to see the impacts that LLIN and IRS campaigns have had on vector densities and malaria incidence. By supporting open access to data, the PRISM team is increasing the utility of the data collected and its impact.

**Table 1:** Data resources, links, and data description for PRISM studies

Study	Data resource and link	Data description
PRISM 1	ClinEpiDB: PRISM ICEMR Cohort	Human clinical and epidemiological data and mosquito abundance data 2011–2017
	PlasmoDB dataset: DS_4267c95a1c	Human serum antibody levels
	VectorBase MapVEu Project: VBP0000647	Mosquito abundance and pathogen type data 2011–2016
	VectorBase MapVEu Project: https://vectorbase.org/vectorbase/app/record/dataset/DS_86aedcf35a	Mosquito abundance data 2015–2017
	VectorBase MapVEu Project: VBP0000648	Mosquito abundance and pathogen type data 2016–2017
PRISM 2	ClinEpiDB: PRISM2 ICEMR Cohort	Human clinical and epidemiological data and mosquito abundance data 2017–2019
	VectorBase MapVEu Project: VBP0000649	Mosquito abundance data 2017–2019
	VectorBase MapVEu Project VBP0000627	Mosquito abundance data 2018

PRISM = Program for Resistance, Immunology, Surveillance, and Modeling of Malaria.

## CONCLUSION

PRISM has provided the infrastructure and expertise required to evaluate the impacts of different malaria control interventions, including IRS, LLINs, chemoprevention, and case management with ACTs on the malaria disease burden in Uganda. These data have informed IRS campaigns, universal LLIN distribution campaigns, and case management strategies, including appropriate coverage, intervals for interventions, choice of insecticide, strategies for IRS discontinuation, and choice of appropriate antimalarial therapies in Uganda. Our enhanced health facility–based surveillance system has generated high-quality data that complement MOH DHIS aggregate data, providing a rich platform for monitoring trends in malaria burden and quantifying the impact of malaria control interventions. Through its comprehensive programs, PRISM has contributed importantly over the past decade to malaria control policy in Uganda. Although the scale-up of proven control interventions resulted in significant reductions in the burden of malaria in Uganda after the turn of the century, since 2015, progress has stalled, similar to other high-burden countries in Africa. Indeed, myriad challenges including the spread of insecticide resistance, the emergence of artemisinin resistance, and the COVID-19 pandemic, have created a precarious situation where accelerated action will be needed to turn the tide and ultimately eliminate malaria in Uganda.
